# The *katG *mRNA of *Mycobacterium tuberculosis *and *Mycobacterium smegmatis *is processed at its 5' end and is stabilized by both a polypurine sequence and translation initiation

**DOI:** 10.1186/1471-2199-9-33

**Published:** 2008-04-04

**Authors:** Claudia Sala, Francesca Forti, Francesca Magnoni, Daniela Ghisotti

**Affiliations:** 1 Dipartimento di Scienze Biomolecolari e Biotecnologie, Università degli Studi di Milano, Via Celoria 26, 20133, Milano, Italy; 2 EPFL-SV-GHI-UPCOL-Station, 15-CH-1015, Lausanne, Switzerland

## Abstract

**Background:**

In *Mycobacterium tuberculosis *and in *Mycobacterium smegmatis *the *furA*-*katG *loci, encoding the FurA regulatory protein and the KatG catalase-peroxidase, are highly conserved. In *M. tuberculosis furA-katG *constitute a single operon, whereas in *M. smegmatis *a single mRNA covering both genes could not be found. In both species, specific 5' ends have been identified: the first one, located upstream of the *furA *gene, corresponds to transcription initiation from the *furA *promoter; the second one is the *katG *mRNA 5' end, located in the terminal part of *furA*.

**Results:**

In this work we demonstrate by in vitro transcription and by RNA polymerase Chromatin immunoprecipitation that no promoter is present in the *M. smegmatis *region covering the latter 5' end, suggesting that it is produced by specific processing of longer transcripts. Several DNA fragments of *M. tuberculosis *and *M. smegmatis *were inserted in a plasmid between the *sigA *promoter and the *lacZ *reporter gene, and expression of the reporter gene was measured. A polypurine sequence, located four bp upstream of the *katG *translation start codon, increased beta-galactosidase activity and stabilized the *lacZ *transcript. Mutagenesis of this sequence led to destabilization of the mRNA. Analysis of constructs, in which the polypurine sequence of *M. smegmatis *was followed by an increasing number of *katG *codons, demonstrated that mRNA stability requires translation of at least 20 amino acids. In order to define the requirements for the 5' processing of the *katG *transcript, we created several mutations in this region and analyzed the 5' ends of the transcripts: the distance from the polypurine sequence does not seem to influence the processing, neither the sequence around the cutting point. Only mutations which create a double stranded region around the processing site prevented RNA processing.

**Conclusion:**

This is the first reported case in mycobacteria, in which both a polypurine sequence and translation initiation are shown to contribute to mRNA stability. The *furA-katG *mRNA is transcribed from the *furA *promoter and immediately processed; this processing is prevented by a double stranded RNA at the cutting site, suggesting that the endoribonuclease responsible for the cleavage cuts single stranded RNA.

## Background

mRNA decay is known to play an important role in the post-transcriptional regulation of gene expression in bacteria. Studies in *Escherichia coli *indicated a possible model, in which the endoribonuclease RNase E binds and cuts the mRNA, followed by RNase II and/or PNPase that degrade the fragments generated by RNase E cleavage [1-5]. The accessibility of the 5' end of an mRNA to RNase E has been shown to be an important mediator of stability in *E. coli *[6,7].

Much less is known about mRNA decay in other bacteria, particularly in gram positive species. Several studies, performed in *Bacillus subtilis *and *Bacillus thuringiensis*, indicated that the 5' terminal leader sequence has a relevant role in mRNA stabilization, either by containing a polypurine rich sequence or by the presence of a predicted 5'-terminal stem-loop structure [8-12]. Extensive mutagenesis was used to define how these two motifs influence mRNA stability and the presence of an RNase E-like ribonuclease, acting on the 5' end, has been suggested. Its action may be blocked by either the stem-loop structure and/or ribosomes located in proximity of the 5' terminus of the transcript [8,12].

In mycobacteria few examples of mRNAs stability control have been described [13-15]. The most interesting is the stability of the DNA gyrase mRNA in *Mycobacterium smegmatis*, which is 5' protected by a stem-loop structure, followed by a Shine-Dalgarno sequence for translation initiation. Disruption of the stem-loop caused loss of transcript stability [15].

In this work we report that the mycobacterial *katG *mRNA originates from specific processing of the longer bicistronic transcript, covering *furA *and *katG*. The *katG *mRNA encodes the catalase-peroxidase, responsible for the degradation of toxic oxygen compounds. In all studied mycobacterial species, the DNA region immediately upstream of *katG *encodes FurA [16-19]. Fur-like proteins are ubiquitous in bacteria, and act as transcriptional repressors that exhibit an iron-dependent DNA binding activity and regulate several genes involved in iron metabolism [20,21]. In Mycobacteria this role is not achieved by FurA, but by the iron dependent transcription regulator (IdeR) which plays a critical role in maintaining the intracellular iron homeostasis [22].

In recent years, genes directly or indirectly controlled by Fur-like proteins have been discovered, leading to the hypothesis that Fur proteins have a wider role in bacterial gene expression [23,24].

**Figure 1:**
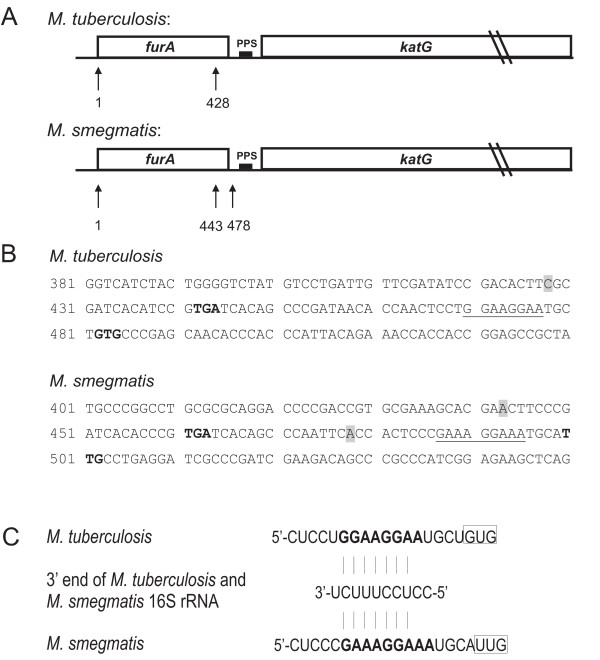
**The *furA*-*katG *region in *M. tuberculosis *and *M. smegmatis***. Panel A. Schematic representation of the *furA*-*katG *region. Genes are indicated by open boxes, the polypurine sequences (PPS) by a black box. The coordinates of the *M. tuberculosis *sequence are arbitrary, with coordinate +1 corresponding to the first codon of *furA *(coordinate 2,156,590 in the reverse strand of the *M. tuberculosis *complete genome sequence deposited in the GenBank:AL123456). Coordinates of the *M. smegmatis *sequence are arbitrary, with coordinate +1 corresponding to one bp immediately upstream of the *furA *start codon (GenBank:NC_008596, [42]). The position of the 5' ends of *katG *mRNA (+428 in *M. tuberculosis*, [25]; 443 and 478 in *M. smegmatis*, [27]) are indicated by vertical arrows below the map. Panel B. Sequence of part of the *furA*-*katG *region of *M. tuberculosis *and *M. smegmatis*. The 5' ends of the *katG *mRNA are highlighted in grey, the stop codon of *furA *and the start codon of *katG *are in bold. The polypurine sequence upstream of *katG *is underlined. Panel C. Complementarity between the 16S rRNA of *M. tuberculosis *and *M. smegmatis *and the PPS upstream of *katG *initiation codon. The PPS is in bold and the translation initiation codon is boxed. The accession number of 16S rRNA of *M. tuberculosis *is GenBank:NC_000962.2 [16] and of *M. smegmatis *is GenBank:NC_008596 [42].

In mycobacteria, the organization of the *furA-katG *region is conserved, suggesting that it may constitute a single operon [16,17,19]. This hypothesis was confirmed by identification of a single *furA-katG *transcript in *Mycobacterium bovis *BCG [18,25]. In apparent contrast, two different 5' ends were identified by S1 mapping: the first coincides with the initiation codon of *furA *and is generated by transcription initiation at the *furA *promoter (Fig. 1A; [18,25,26]); the second is 54 bp upstream of *katG *translation start codon. Master *et al*. [25] proposed that a *katG *promoter was present in this region (Fig. 1A and 1B).

In *M. smegmatis *specific transcripts for *furA *and *katG *were identified by Northern blotting, but a single transcript encompassing both *furA *and *katG *genes was not detected [27]. Specific 5' ends were mapped by primer extension analysis [26,27]: the first (coordinate 1 in Fig. 1A) was due to transcription initiation from the *furA *promoter; two additional 5' ends were mapped upstream of *katG*, the first one, corresponding to coordinate 443, falls in the terminal part of the *furA *gene; the second is less intense and corresponds to coordinate 478, located in the *furA-katG *intergenic region. No specific promoter sequences were identified upstream of either 443 or 478.

In this work we addressed the question whether the *katG *5' ends were generated by transcription initiation or by mRNA processing. Our results support the latter alternative. Furthermore, a polypurine sequence and translation initiation are both involved in *katG *mRNA stability.

## Results

### The 5' end of *katG *mRNA is not generated by initiation of transcription

**Figure 2:**
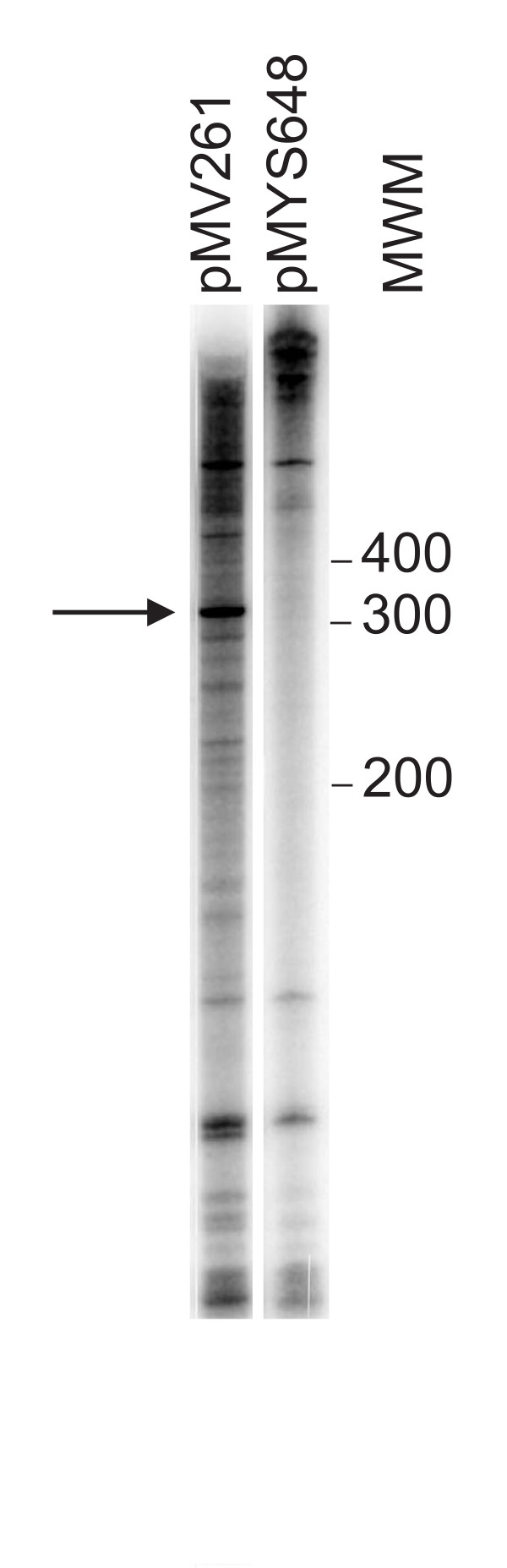
**In vitro transcription**. In vitro transcription was performed with 10 μg of crude *M. smegmatis *extract in the presence of [α-^32^P]-CTP. The templates were plasmid pMV261, which carries the *M. bovis *BCG *hsp60 *promoter, and plasmid pMYS648, in which the 437–552 *M. smegmatis *region has been substituted to the *hsp60 *promoter region. The samples were run on a 5% denaturing polyacrylamide gel. Molecular weight markers (MWM) run in the same gel are indicated on the right. The about 300 bp transcript synthesized by pMV261 is indicated by an arrow.

In a previous work, Milano *et al*. [27] reported the presence of a promoter region for *katG *expression in *M. smegmatis*, located in the terminal part of the upstream *furA *gene. In order to demonstrate the presence of this promoter, we amplified the *M. smegmatis *region that overlaps the 5' ends upstream of *katG *(coordinate 437–552) and cloned it in plasmid pMV261, substituting the *hsp60 *promoter region (pMYS648). In vitro transcription was performed in the presence of [α^32^P]CTP and *M. smegmatis *cell extract, comparing the transcripts synthesized by pMV261, from the constitutive *hsp60 *promoter, and pMYS648, from the potential *katG *promoter (Fig. 2). pMV261 produced an RNA of about 300 nt that may represent the RNA starting from the *hsp60 *promoter and terminating at the transcription terminator downstream. On the contrary, no transcript of the expected size for an mRNA starting at the hypothetical *pkatG *and terminating at the transcription terminator (about 200 nt) was detected when pMYS648 was used, suggesting that no promoter was present in this region. This data is in agreement with the absence of any promoter consensus sequences upstream of the 5' ends.

In order to exclude that a *katG *promoter was present in this region, we performed an RNA polymerase Chromatin Immunoprecipitation assay (RNApol-ChIP) on *M. smegmatis *mc^2^155. After rifampicin treatment in order to block RNA polymerase on promoter sequences [28], formaldehyde cross-linking, and sonication, the DNA-RNA polymerase complexes were immunoprecipitated with mouse monoclonal antibodies to the β subunit of *E. coli *RNA polymerase, as described in the Methods section. As rifampicin inhibits transcript elongation, we expected RNA polymerase to be trapped at any potential promoter. The precipitated DNA was then analyzed by quantitative RT-PCR to determine the amount of recovered DNA.

**Figure 3:**
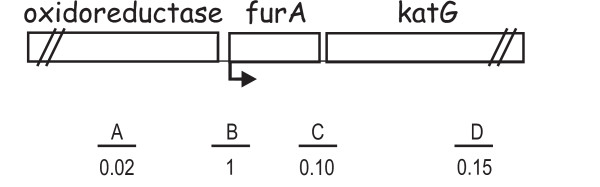
**Results of RNAPol-ChIP experiments**. Schematic representation of the oxidoreductase-*furA*-*katG *region of *M. smegmatis*. The genes are indicated by boxes and the arrow corresponds to the *furA *promoter. The lines below the map correspond to different regions amplified by a pair of oligonucleotides. Fragment A: -689/-492; B: -81/91 (*furA *promoter); C: 374/557; D: 1155/1344. Below the lines, the values of the ratio of each fragment relative to the *pfurA *DNA.

Specific oligonucleotides were used to amplify different 150–200 bp DNA fragments of the *furA-katG *region (fragments A, B, C, and D in Fig. 3). The amplified regions with the indication of the ratio relative to the amount of the fragment containing the *pfurA *promoter (fragment B) are reported in Fig. 3. Compared to fragment B, all the other regions were recovered with low frequency: in particular, the region where the potential *katG *promoter had previously been located (fragment C) was recovered with a relative value of 0.10. Thus, we concluded that a promoter was not present in this region and that the *katG *mRNA 5' end was produced by processing of a longer transcript.

### The polypurine sequence preceding the *katG *start codon stabilizes the downstream transcript

In *Bacillus subtilis *and *Bacillus thuringiensis *polypurine sequences (PPS) in the 5' terminal part of several transcripts were reported to increase mRNA stability [9-11]. By sequence analysis of the region immediately upstream of *katG *in *M. tuberculosis *and *M. smegmatis *we identified a PPS four bases upstream of the *katG *translation start codon (Fig. 1B; GGAAGGAA at coordinates 470–477 in *M. tuberculosis*; GAAAGGAAA at coordinates 487–495 in *M. smegmatis*). We tested whether the PPS in the mycobacterial *katG *mRNA was also able to stabilize the transcript.

**Figure 4:**
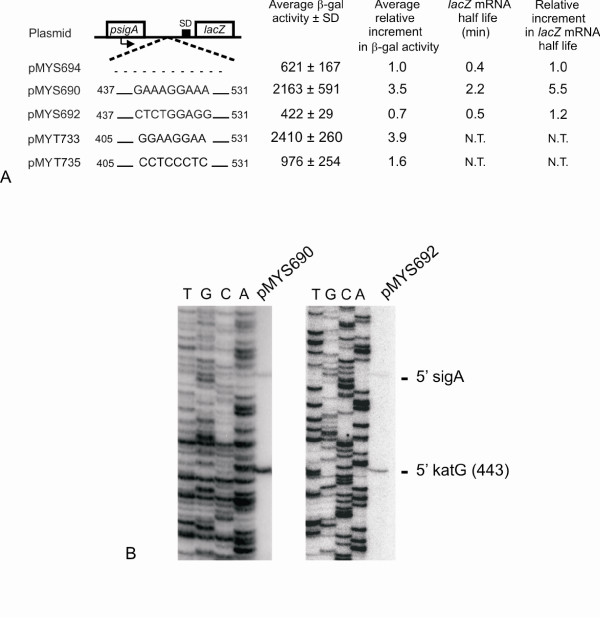
**Effect of the polypurine sequence on RNA stability**. Panel A. pMYS694 carries the *M. smegmatis *-195/+27 DNA region (where +1 is the transcription initiation site from *psigA *[29]), containing the *sigA *promoter, upstream of *lacZ *in pSM128. The *lacZ *gene is preceded by its ribosome binding site (44 nt from the cloning site). pMYS690 carries the 437–531 region of *M. smegmatis *downstream of *psigA*, containing the wild type PPS (GAAAGGAAA); pMYS692 carries the same region with the indicated mutations in the PPS (CTCTGGAGG). pMYT733 and pMYT735 carry the 405–531 region of *M. tuberculosis *downstream of *psigA*. In pMYT733 carries the wild type PPS (GGAAGGAA), whereas in pMYT735 the PPS was substituted by CCTCCCTC. Beta-galactosidase activity and the half life of the *lacZ *mRNA were determined as indicated in the Methods section. Average beta-galactosidase activity in Miller Units of three to eight different replicas ± standard deviation is reported. The average relative increment in beta-galactosidase activity and in *lacZ *mRNA half life are also indicated. N.T. = not tested. Panel B. RNA was extracted from *M. smegmatis *strains mc^2^155(pMYS690) and mc^2^155(pMYS692), and primer extension performed with oligonucleotide 809, internal to *lacZ*, as described in Methods. The DNA sequence obtained with the same oligonucleotide on plasmid pMYS690 was run in the same gel.

An integrative plasmid was constructed, pMYS694, that carries the *M. smegmatis sigA *promoter [29] upstream of the *lacZ *reporter (Fig. 4A). Then, either the 437–531 region of *M. smegmatis *(pMYS690) or the 405–531 region of *M. tuberculosis *(pMYT733) were cloned between the *sigA *promoter and the *lacZ *gene. These regions cover the terminal part of the *furA *gene, the *furA*-*katG *intergenic region with the wild type PPS and the first codons of *katG *(Fig. 4A). Two mutant plasmids were also constructed, in which the PPS sequence was mutagenized (pMYS692 and pMYT735 carry CTCTGGAGG and CCTCCCTC, respectively; see Fig. 4A).

*M. smegmatis *mc^2^155 was transformed, and beta-galactosidase activity measured. As reported in Fig. 4A, beta-galactosidase activity expressed by pMYS694 was increased 3.5-fold in pMYS690 and 3.9-fold in pMYT733, which carry the wild type PPS. On the contrary, beta-galactosidase expressed from pMYS692 and pMYT735, carrying the mutated PPS, did not change significantly compared to the control pMYS694 (0.7 and 1.6 fold, respectively).

Furthermore, we measured the half-life of *lacZ *mRNA from the plasmids carrying *M. smegmatis *fragments. The mc^2^155 strains carrying pMYS694/690/692 were treated with rifampicin (see Methods) to block transcription and the RNAs were extracted from the cultures at 0, 2, 4, 10, and 30 minutes after the addition of the antibiotic. Quantitative RT-PCR was performed in order to measure the amount of RNA at the different time points and to evaluate the half lives in the three strains. The half life of the *lacZ *mRNA was increased from 0.4 minutes in pMYS694 to 2.2 minutes in pMYS690 (5.5 fold increase), whereas no substantial increase was observed in pMYS692, which carries the mutated PPS. These results suggest that the identified PPS is able to stabilize the *katG*-*lacZ *mRNA. Moreover, we observed a good correlation between the beta-galactosidase activity and the *lacZ *mRNA stability.

Primer extension analysis was performed on the RNA extracted from mc^2^155(pMYS690) and (pMYS692) (Fig. 4B). Two bands were detected in pMYS690: one corresponding to the previously mapped 5' end of *sigA *[29], and the other corresponding to the 5' end of *katG *(443 [27]) in *M. smegmatis*. The band at 443 appears very strong, in accordance with the stabilizing effect of the downstream PPS. The two signals were also present in pMYS692, but in this case the 443 band was visibly fainter, according to the decreased stability caused by the mutated PPS.

### Relevance of translation in *katG *mRNA stability

In other gram positive bacteria it has been shown that ribosome binding and ternary complex formation are responsible for the PPS mediated stability, whereas translation appears not to be involved [11,31,32]. On the other hand, in many *E. coli *RNAs translation seems to contribute directly to transcript stability [33-35].

**Figure 5:**
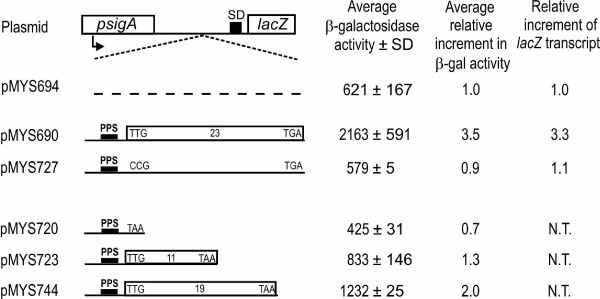
**Role of translation in mRNA stability**. Beta-galactosidase expression of *M. smegmatis *mc^2^155 transformed with the different plasmids, derived from pMYS694 (in the legend to Fig. 4A), by inserting *M. smegmatis katG *DNA regions of different length, downstream of the *sigA *promoter. All the plasmids carry the same region from 437 to the start codon of *katG *(coordinate 499, see Fig. 1), and differ for the length of the following open reading frame: pMYS690 and pMYS727 carry the same DNA region, but the *katG *translation start codon TTG has been substituted in pMYS727 with a CCG codon; pMYS720 is mutated in the start codon of *katG *(TTG to TAA codon); pMYS723 and pMYS744 carry 11 and 19 codons of *katG*, respectively. Strain mc^2^155 was transformed with the plasmids and beta-galactosidase expression was measured, as described in Methods. Average beta-galactosidase activity in Miller Units of three to eight different replicas ± standard deviation is reported. The average relative increment in beta-galactosidase activity is also indicated. The amount of *lacZ *transcript was evaluated by quantitative RT-PCR. N.T. = not tested.

In order to evaluate the role of translation in increasing mRNA stability in our system, we constructed several plasmids in which DNA fragments of *M. smegmatis *were inserted in pMYS694 between the *sigA *promoter and the *lacZ *reporter gene (Fig. 5). pMYS690 carries an *M. smegmatis *region that starts from coordinate 437 and extends to the first 23 codons of *katG*, followed by a translation stop codon (TGA). pMYS727 carries the same DNA fragment, but the *katG *translation start codon was mutagenized to CCG.

In all these constructs translation of the downstream *lacZ *gene proceeds independently, by ribosome binding to the *lacZ *ribosome binding site and starting translation from its ATG start codon. Thus, beta-galactosidase activity and the stability of the *lacZ *transcript can be compared in the different constructs.

Both the amount of beta-galactosidase and of *lacZ *transcript were increased more than 3 fold in pMYS690 compared to pMYS694, whereas in pMYS727 relative expression was even lower than in the control. These results suggest that, beside the PPS, translation of the *katG *open reading frame is also directly involved in transcript stability.

Other plasmids were constructed in which the length of the *katG *open reading frame was varied: in pMYS720 a stop codon substitutes the *katG *start codon (TTG to TAA); in pMYS723 and pMYS744 the translation stop codon is located after 11 and 19 codons, respectively. We measured beta-galactosidase activity as an indication of the *lacZ *mRNA stability. pMYS720 expressed beta-galactosidase activity to a level similar to the pMYS694 control, confirming the relevance of translation for transcript stability. An increased expression of beta-galactosidase could be observed in pMYS723 and in pMYS744, in which translation can proceed for 11 and 19 codons, respectively. These data indicate that translation of about twenty or more codons is necessary for achieving full transcript stability.

### A single stranded mRNA region is necessary for the correct processing of the *katG *mRNA

**Figure 6:**
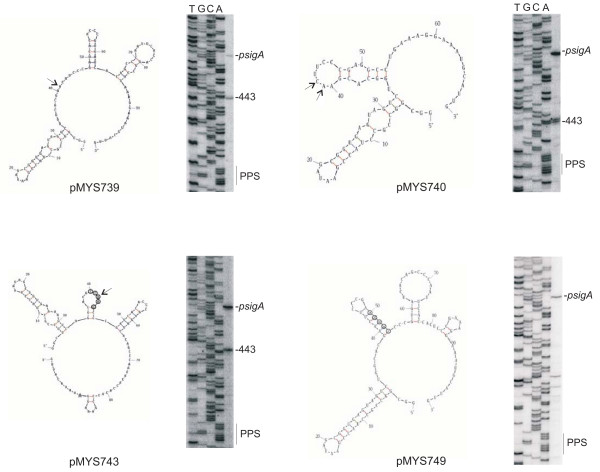
**Structure prediction and primer extension on mRNAs synthesized by different plasmids**. The structure of the transcripts starting at the *sigA *promoter in pMYS739, pMYS740, pMYS743 and pMYS749 were obtained with the m-fold structure prediction program of Zuker [41] and primer extension analyses were performed on RNAs extracted from mc^2^155 transformed with the different plasmids, with oligonucleotide 809. Sequence reactions obtained with the same oligonucleotide were run in the same gel. The position of the *sigA *promoter, the 443 processing site and the PPS region are indicated on the right. The arrows indicate the position of the cut on the sequence structure. The mutations are circled.

To characterize the requirements for the processing of the *katG *mRNA 5' end, several plasmids were constructed by insertion of *M. smegmatis *DNA regions from 437 to 552, downstream of *psigA*: pMYS739 carries the wild type sequence, pMYS740 carries a 30 bp internal deletion (from 450 to 480), in pMYS743 five bp flanking the processing site have been substituted, and in pMYS749 five bp were substituted in order to allow the formation of a double stranded region corresponding to the processing site (see Fig. 6). For each plasmid the predicted structure of the RNA from *sigA *is reported in Fig. 6. RNAs were extracted from mc^2^155 strain transformed with the plasmids and primer extension was performed with oligo 809, internal to the downstream *lacZ *gene. The results indicated that in the wild type pMYS739 two signals were detectable: one corresponding to the 5' end due to *sigA*, the second corresponding to the processing site at 443. The only small effect of the 30 bp deletion in pMYS740 was the presence of a double signal at 442 and 443. The transcript of pMYS743, which carries a different sequence around the processing site, is normally processed at 443, whereas the transcript of pMYS749, in which a double stranded region is created, was no more correctly processed. In order to confirm that a double stranded RNA around 443 could not be processed, we constructed an other plasmid in which different bases were substituted in order to create a double stranded RNA, and also in this case processing at 443 did not occur (data not shown). These data indicate that the ribonuclease responsible for the processing needs a single stranded RNA region for cutting.

## Discussion

### The 5' end of the *katG *transcript in *M. tuberculosis *and *M. smegmatis *is a processing site

In a previous work [27] we suggested the presence of a promoter for *M. smegmatis katG *expression in the terminal part of the *furA *gene and we identified two 5' ends at coordinate 443 and 478. However, in this work we tried without success to use the cloned fragment for in vitro transcription: no RNA could be produced by pMYS648, in which the *M. smegmatis *437–552 region has been cloned. Moreover, neither promoter consensus sequences were present upstream of 443 nor this region could be recovered by RNA-polymerase ChIP assays. Thus, we concluded that the 5' end is not due to transcription initiation rather to processing of a longer transcript.

In *M. tuberculosis *a longer transcript, starting from *pfurA*, has been observed [18,25]. In *M. smegmatis *we were unable to detect a polycistronic *furA*-*katG *transcript by RT-PCR experiments [27]. This suggests that immediate processing may follow *furA*-*katG *transcript synthesis in *M. smegmatis*.

In the constructs in which the *sigA *promoter region has been cloned immediately upstream of the *M. smegmatis *fragment (pMYS690 and pMYS692), we could demonstrate the presence of a longer RNA, starting at *psigA*. Primer extension analysis also revealed the presence of a 5' end at 443, that is the same position in which the *M. smegmatis furA-katG *mRNA is cut. However, no primer extension signal was observed at 478. In fact, this latter 5' end was no longer detected in many primer extension experiments performed with RNAs extracted from mc^2^155 (unpublished data). We suggest that this signal, always less intense than the 5' end at 443, may arise by specific pausing of the reverse transcriptase.

### Role of a polypurine sequence in *katG *mRNA stability

By cloning downstream of the *sigA *promoter the *M. tuberculosis *or the *M. smegmatis *regions that cover the *katG *gene translation start point, beta-galactosidase expression and *lacZ *mRNA half life were increased more than threefold. These regions contain a PPS four bp upstream of the *katG *translation start codon. Mutagenesis of the PPS abolished both effects, thus suggesting the central role of this sequence in transcript stability.

The complementarity of the PPS to the 16S rRNA sequence (Fig. 1C) indicates that it could act as the *katG *ribosome binding site. Mutations altering the complementarity to the 16S rRNA decreased the amount of the *lacZ *transcript, suggesting that ribosome binding protects the RNA from degradation.

The role of ribosome binding sites in mRNA stabilization has been demonstrated in many gram-positive and gram-negative bacteria. It was shown that many mRNAs, like *lacZ *and *ompA *of *E. coli *[33,34,36], *aprE*, *gsiB *and *ermC *of *B. subtilis *[31,32,37,38], *cryIIIA *of *B. thuringiensis *[11], can be significantly destabilized by mutations in the ribosome binding site that interfere with ribosome binding by reducing its complementarity to 16S rRNA.

This is the first example in mycobacteria, in which a polypurine rich sequence is able to confer stability to the downstream RNA likely by ribosome binding.

### Translation is required for stabilization

Most examples of mRNA stability mechanisms derived from gram-positive bacteria seem to depend on ribosome binding, but not on translation [9-12,31,32,38]. On the other hand, several examples in *E. coli *indicate that translation has also a role [33-36,39,40].

We could demonstrate that in our mycobacterial system translation is required for stabilization, and more than 20 codons are needed for conferring full stability to the transcript. Thus, we suggest that the ribonuclease that processes the transcript is impaired both by ribosome bound to the PPS and by translation of the first part of *katG*.

### Regulation of KatG expression and processing of the *katG *transcript

In previous works [25,27], by means of two different reporter systems, *katG *expression was suggested to be originated from specific transcription initiation. Considering our new findings, indicating the absence of any promoter and the presence of a stabilizing sequence upstream of the *katG *start codon, a new explanation should be given to previous results. We hypothesize that the reporter activity could be explained by the presence of an unknown promoter upstream of the cloning site in the vector sequence. Transcription starting from this promoter may produce a transcript that, upon processing at 443, is stabilized by both the polypurine sequence and translation initiation. This led to the expression of the downstream reporter gene. Thus, a reporter gene assay, usually used to identify promoter sequences, led us to detect an RNA stabilizing sequence.

Therefore, in agreement with what has been suggested by Pym *et al*. [18], in *M. tuberculosis *and in *M. smegmatis *transcription of the *katG *gene may originate from the upstream *pfurA *promoter. A single transcript containing *furA *and *katG *is synthesized and processed to originate two different transcripts, one covering *furA*, the second covering *katG*. The latter is stabilized by the PPS and translation of the first codons of *katG*. In conclusion, expression of *katG *appears to be under the direct control of the FurA protein that controls transcription initiation at *pfurA *[26].

Although the transcript is processed both in *M. tuberculosis *and in *M. smegmatis*, the efficiency of the processing event differs. This may be the consequence of a better substrate in *M. smegmatis *and/or a more active RNAse. It would be possible to replace the *M. smegmatis furA*-*katG *intergenic region by the corresponding *M. tuberculosis *region and test whether a single mRNA for both genes can be found. If this occurs, one can conclude that the substrate makes the difference. On the contrary, if the transcript is completely processed, *M. smegmatis *RNAse is more active.

The secondary structure of the transcripts covering the *M. smegmatis furA-katG *region were obtained by mfold-structure prediction program of Zuker [41]. The structure of the region surrounding the processing sites is single stranded both in the transcripts starting at the *furA *promoter (not shown) and in the RNAs starting at *psigA *(Fig. 6; pMYS739). Moreover, mutagenesis of this region, either by deletion of about 30 bp or by substitution of the bases surrounding the processing site did not prevent processing at 443. On the contrary, a double stranded RNA region impaired the processing, suggesting that the RNase involved in the processing of the *furA-katG *transcript requires single stranded RNA.

Several endoribonucleases have been identified in *M. tuberculosis *and in *M. smegmatis *complete genome sequence by homology with known enzymes in other bacteria [16,42]. Experiments are in progress to identify which mycobacterial ribonuclease is responsible for this processing.

## Conclusion

This is the first reported case in mycobacteria, in which both a polypurine sequence and translation initiation are shown to contribute to mRNA stability. The *furA-katG *mRNA is transcribed from the *furA *promoter and likely processed by a single stranded RNAse.

## Methods

### Bacterial strains and culture conditions

*M. smegmatis *mc^2^155 [43] was grown in LD medium [44] containing 0.2% (vol/vol) glycerol and 0.05% (vol/vol) Tween 80 and supplemented when necessary with spectinomycin (100 μg/ml). *Escherichia coli *DH10B [45] was used as host strain for cloning and plasmid propagation.

### Plasmids

pMV261 is a multicopy mycobacterial vector, carrying the promoter region of the *M. bovis *BCG *hsp60 *gene about 300 bp upstream of the *rrnABt1 *transcriptional terminator [46]. pMYS648 was constructed by inserting the PCR amplified *M. smegmatis *437–552 region into pMV261, digested *Xba*I – *Msc*I, replacing the *hsp60 *regulatory region.

pSM128 is an integrative vector carrying the *lacZ *reporter gene [47]. Plasmids derived from pSM128, containing DNA fragments from either *M. tuberculosis *or *M. smegmatis*, were obtained by PCR amplification of the regions, digestion and cloning in the *Sca*I site, upstream of the *lacZ *reporter gene. Some of the mutated DNA fragments were obtained by the overlap extension technique [48]. The sequence of the oligonucleotides used for plasmid constructions will be provided upon request. All the inserted fragments were sequenced.

pMYS739, pMYS740, pMYS743 and pMYS749 carry the *psigA *promoter [29] and the 437–552 region of *M. smegmatis *upstream of the *lacZ *reporter gene. pMYS740 carries the 437–552 region of *M. smegmatis *with a deletion of the 450–480 region; pMYS743 carries the 437–552 region of *M. smegmatis *with mutagenesis of the nucleotides in position 442–446 (from AACTT to CCAAG) and pMYS749 carries the 437–552 region of *M. smegmatis *with mutagenesis of the nucleotides in position 452–456 (from TCACA to AGTTC).

### Preparation of crude extracts

Crude extracts of *M. smegmatis *were prepared from 100 ml log phase cultures (OD_600 _= 0.8). The cells were pelleted, washed in 20 mM TrisHCl pH 8, 10 mM MgCl_2_, 0.1 mM EDTA, 0.1 mM DTT, 50 mM KCl, and resuspended in 500 μl of 20 mM TrisHCl pH 8, 10 mM MgCl_2_, 0.1 mM EDTA, 0.1 mM DTT, 50 mM KCl, 1 mM PMSF, 10% glycerol. The cells were lysed by sonication (2 × 20 seconds at 40% amplitude). The cell lysate was spun for 10 min at 15000 × g and the supernatant (about 10 mg/ml) was used as the crude extract.

### In vitro transcription assay

Approximately 10 μg of crude extract and 4 μg of CsCl-banded plasmid DNA were incubated for 10 min at 37°C in assay buffer (40 mM Tris pH 7.9, 6 mM MgCl_2_, 2 mM spermidine, 10 mM NaCl, 100 μg/ml bsa, 20 units of rnasin), followed by addition of UTP, ATP and GTP at 0.4 mM, CTP at 0.2 μM, and 5 μCi [α-^32^P]-CTP per 20 μl reaction. After incubation for 30 min at 37°C, 80 μl of 200 mM EDTA and 20 μg of yeast tRNA were added and the reaction mixture was extracted with phenol-chloroform and ethanol precipitated. The pellet containing the labelled RNA was resuspended in sample buffer and analysed on a 5% denaturing polyacrylamide gel.

### Rifampicin treatment

For RNA half-life measurements, cultures were grown up to OD_600 _= 0.8, treated with rifampicin (500 μg/ml), and, at different time points, RNA was isolated as described below.

### RNA extraction

Fifteen ml of *M. smegmatis *mc^2^155 cultures carrying the different plasmids (OD_600 _= 0.8) were pelleted and resuspended in 100 μl of Tris-EDTA buffer; 75 μl of RNA lysis buffer (4 M guanidinium thiocyanate, 0.01 M Tris pH 7.5, 0.97% β-mercaptoethanol) were added and the suspension was sonicated (20 seconds at 40% amplitude). The RNA was then purified using the SV Total RNA Isolation System according to the manufacturer's protocol (Promega).

### Primer extension

Twenty μg of RNA extracted as described above were used in Primer Extension analysis [49] with oligonucleotide 809 (5'-TGCGTTTGTTTGCACGAACC-3') internal to *lacZ*.

### Beta-Galactosidase assay

Independent cultures of *M. smegmatis *mc^2^155 carrying the indicated plasmids were grown at 37°C to OD_600 _= 0.8. Cells were collected by centrifugation, resuspended in 500 μl of TEDP (0.1 M Tris-HCl, 1 mM EDTA, 1 mM DTT and 1 mM PMSF), and disrupted by sonication (2 pulses of 20 seconds and 40% amplitude). Beta-galactosidase activity of the extracts was measured as described in Miller [50]. The enzyme activity was expressed as nanomoles of *o*-nitrophenol-beta-galactopyranoside converted to *o*-nitrophenol minute^-1 ^milligram of protein^-1^. Each experiment was performed three times.

### Quantitative RT-PCR

Ten μg of RNA extracted as described above were retrotranscribed with Superscript II Reverse Transcriptase (Invitrogen) using random primers. Double stranded DNA binding dye iQ SYBR Green Supermix (Bio-Rad) in an iCycler iQ real time PCR detection system from Bio-Rad was used to quantify the number of target cDNA molecules in the different samples. Each reaction was run in triplicate and the melting curves were constructed. The relative expression of *lacZ *mRNA was determined using the Δ (Δ Ct) method [51], with either the 16S rRNA or the *sigA *mRNA as standard.

### RNA pol-ChIP

Ten ml *M. smegmatis *culture was grown up to OD_600 _= 0.8, rifampicin was added to a final concentration of 500 μg/ml and the culture incubated for 5 minutes at 37°C with shaking. The culture was then fixed with 1% formaldehyde at room temperature for two minutes under gentle shaking and the reaction stopped by addition of glycine (final concentration 0.125 M) for 10 minutes with gentle shaking. Cross-linked cells were pelleted by centrifugation, washed twice in PBS and once in STET (150 mM NaCl, 10 mM Tris-HCl pH 8, 10 mM EDTA, 0.25% Triton X-100), and finally resuspended in 2 ml of TE (10 mM Tris-HCl pH 8, 1 mM EDTA). The sample was sonicated on ice with 20 pulses of 20 seconds and 40% amplitude. The average DNA fragment size obtained was approximately 0.5 kb. After sonication insoluble debris was removed by centrifugation and the extract precleared with 40 μl 50% protein G-agarose slurry (Sigma, previously blocked with 100 μg/ml lambda DNA, 500 μg/ml tRNA, 1 mg/ml BSA) for 3 h at 4°C. 100 μl of the precleared extract were saved as *input *fraction. 900 μl were incubated under rotation overnight at 4°C, with 10 μl of 8RB13 mouse monoclonal antibodies to β subunit of *E. coli *RNA polymerase (Neoclone, cross-reacts with RNA polymerases of many others prokaryotes) in 1× RIPA buffer (140 mM NaCl, 10 mM Tris HCl pH 8, 1 mM EDTA, 1% Triton X-100, 0.1% SDS, 0.1% Na deoxycholate) and RNA polymerase-DNA complexes were immunoprecipitated with 50 μl 50% protein G-agarose slurry for 4 h under rotation at 4°C. The immunocomplex was recovered by centrifugation, washed four times with 1× RIPA buffer, once with LiCl buffer (250 mM LiCl, 10 mM Tris-HCl pH 8, 1 mM EDTA, 0.5% NP-40, 0.5% Na deoxycholate), twice with TE, and resuspended with 100 μl TE (IP fraction). 900 μl of the precleared extract were treated as above but in the absence of the antibodies (*no ab *fraction). The DNA of the *input*, IP and *no ab *fractions was recovered by treatment for 30 min at 37°C with RNase A (20 μg/ml) and overnight digestion with 50 μg/ml proteinase K in 0.5% SDS at 37°C. Crosslinking was reversed for 6 h at 65°C. DNA was extracted once with phenol/chlorophorm, ethanol precipitated and resuspended in 50 μl TE.

The DNA of the samples (1:10 dilution) was analyzed by quantitative PCR, as described above. Four primer pairs, useful to amplify different fragments in the *furA/katG *region of *M. smegmatis*, were used. The coordinates of the DNA fragments are reported in the legend to Figure 3.

The amount of the amplified DNA was calculated using the standard curve of each primer pair. The results of the *no ab *sample were subtracted from those of the IP sample, the resulting figures divided by their corresponding *input *and finally the result of each primer pair divided with that of the *pfurA *region pair.

### Folding and hybridization prediction

The RNA sequence of the transcripts synthesized by pMYS739, pMYS740, pMYS743 and pMYS749 have been analyzed by the mfold program of Zucker [41].

## Authors' contributions

CS carried out the molecular genetic studies and participated to the design of the manuscript. FF carried out the ChIP assay, in vitro transcription, and quantitative RT-PCR. FM constructed the PPS mutations, tested their effect on beta-galactosidase expression, and performed the RNA structure with M-fold program. DG conceived with the study, and partecipated in its design and coordination and helped to draft the manuscript. All authors read and approved the final manuscript.
